# G Protein-Coupled Receptor Kinase 4 Is a Novel Prognostic Factor in Hepatocellular Carcinoma

**DOI:** 10.1155/2022/2628879

**Published:** 2022-06-20

**Authors:** Yunxiu Luo, Ziyi Wang, Shengjun Xiao, Ruirui Li, Xiaoshan Jiang

**Affiliations:** ^1^Xiangya Hospital, Central South University, Changsha, Hunan 410008, China; ^2^Cell Signaling Laboratory, Guilin Medical University, Guilin, Guangxi 541004, China; ^3^Department of Pathology, Guilin, Guangxi 541100, China; ^4^Breast Center and, Guilin, Guangxi 541100, China; ^5^Guangxi Health Commission Key Laboratory of Glucose and Lipid Metabolism Disorders, The 2nd Affiliated Hospital of Guilin Medical University, Guilin, Guangxi 541100, China

## Abstract

**Purpose:**

We previously reported that G protein-coupled receptor kinase (GRK) 4 halts cell cycle progression and induces cellular senescence in HEK293 cells. The present study was aimed at assessing the prognostic value of GRK4 in hepatocellular carcinoma (HCC).

**Methods:**

GRK4 expression was detected by immunohistochemistry in paired tumoral and peritumoral tissues of 325 HCC patients. One hundred and twenty-six patients from Western China were utilized as a training cohort to develop a nomogram, while 86 patients from Eastern China were used as a validation cohort. The proliferation and migration of lentiviral-GRK4 expressing HepG2 cells were determined by MTT and wound healing assays.

**Results:**

GRK4 was differentially expressed in HCC tissues. Tumoral GRK4 intensity, tumor type, and T stage were independent prognostic factors and used to form a nomogram for predicting overall survival (OS), which obtained a good concordance index of 0.82 and 0.77 in training and validation cohort, respectively. The positive and negative prediction values with nomogram were, respectively, 83% and 75% in training cohort and 100% and 52% in validation cohort. Patients with nomogram scores > 32 and 78 showed high risk for OS. Proliferation and motility capabilities were significantly restrained in GRK4-overexpressing HCC cells. *Discussion*. Low GRK4 expression in HCC tumor tissues indicates poor clinical outcomes. A prognostic nomogram including tumoral GRK4 expression would improve the predictive accuracy of OS in HCC patients. We also demonstrated that GRK4 overexpression inhibits proliferation and migration of HCC cells. The molecular mechanism underlying is worth further study.

## 1. Introduction

Hepatocellular carcinoma (HCC) is the sixth most prevalent cancer and the third leading cause of cancer-related mortality worldwide [1]. Although hepatectomy, along with other therapies including radiation, radiofrequency ablation (RFA), and transcatheter arterial chemoembolization (TACE), have provided survival benefits for HCC patients, the frequent intrahepatic recurrence and extrahepatic progression remain challenging [2]. Systemic treatment using multiple lines of the multitargeted tyrosine kinase inhibitor sorafenib or regorafenib has demonstrated an increase in median survival of advanced patients [3, 4]. In general, the 5-year survival rate for HCC is still very low (i.e., <13% in China and <20% in the United States) [5, 6]. The TNM staging is a concise system widely used to predict the prognosis for postoperative cancer patients, but its practicability is limited for HCC since the patients with same TNM stage often have different clinical outcomes [2]. CD4+ CD25+ regulatory T cells (Tregs) play a critical role in maintenance of hepatic immune tolerance. Tregs suppress the antitumor immune response and promote HCC invasion via TGF-*β*1-dependent mechanisms [7]. The frequency of Tregs in both circulation of HCC patients and in HCC tumor tissues was higher than in healthy controls and peritumoral tissues [8]. Several studies have reported the prognostic value of Tregs, but the results are controversial [9]. An applicable prognostic system for HCC remains to be determined.

G protein-coupled receptor kinases (GRKs) are a family of serine/threonine kinases. In mammals, seven GRK members have been identified so far. They are grouped into three subfamilies: the GRK1 subfamily (rhodopsin kinase subfamily, GRK1 and GRK7), the GRK2 subfamily (*β*-adrenergic receptor kinase subfamily, GRK2 and GRK3), and the GRK4 subfamily (GRK4, GRK5, and GRK6) [10, 11]. GRKs are involved in a wide range of cellular physiological and pathological activities by phosphorylation of the activated G protein-coupled receptors (GPCRs) or through non-GPCR phosphorylation [10, 12, 13]. Certain GRK signaling pathways are closely related to the occurrence and progression of tumors [14, 15]. Immunohistochemistry staining (IHS) showed that the expressions of GRK2 and GRK3 were significantly lower in tumor than in adjacent tissues, correlated with disease-free survival and overall survival (OS) in HCC patients [16, 17], while the GRK6 might mediate a tumor growth signaling [18].

The human GRK4 gene is composed of 16 exons and four splice variants (GRK4*α*/*β*/*γ*/*δ*) have been identified [19]. GRK4 has been the least understood member of the GRKs. Unlike the widespread expressions of GRK2, GRK3, GRK5, and GRK6, GRK4 is limited to the testes, myometrium, kidney, and brain [20]. The biological function of GRK4 involves the desensitization of LH, FSH, mGlu, GABA (B), dopamine D1, and angiotensin type 1 receptors [21, 22]. GRK4 has been linked to the etiopathogenesis of essential hypertension [23, 24]. Two studies showed heterogenous expression for the differential GRK4 isoforms in human granulosa cell tumors and invasive breast cancer [25, 26]. We recently reported that exogenous expression of GRK4*α* halts cell cycle progression and induces cellular senescence in HEK293 cells [27], which involves in pathways of cellular development, proliferation, apoptosis, aging, and cell death [28].

In this study, distribution of the full-length GRK4 (GRK4*α*, referred as GRK4 in this manuscript) in tumor and peritumor tissues and its significance in the prognosis of patients with HCC were investigated. We found that GRK4 is differentially expressed in HCC tumor tissues and low expression of GRK4 is associated with the poor prognosis. A prognostic nomogram including tumoral GRK4 expression would improve the predictive accuracy of OS in HCC patients. To our knowledge, this is the first nomogram model based-on GRK4 risk stratification in cancer.

## 2. Methods

### 2.1. Patients and Tissue Samples

Data collected from patients who underwent hepatectomy and were histologically diagnosed with primary HCC at The Second Affiliated Hospital of Guilin Medical University (2^nd^ AHGMU, Guilin, China) between January 2012 and March 2017. The study was approved by the Institutional Ethics Committee of 2^nd^ AHGMU. The inclusion criteria were as follows: (1) OS was not less than three months; (2) patients received tumor resection; and (3) tumor tissues and corresponding peritumor tissue samples were available. Patients who had any preoperative anticancer treatments, extrahepatic metastases or incomplete clinical data, or undergoing liver resection after intrahepatic recurrence or metastasis were excluded. The HCC tissues and adjacent healthy tissues were formalin-fixed and paraffin-embedded for construction as a tissue microarray (TMA). The paired HCC tissue TMA from our hospital was used for the training cohort to develop the nomogram, and another TMA (TMA HLivH180Su10; Shanghai Outdo Biotech), which was obtained from the National Engineering Centre for Biochip at Shanghai (Shanghai, China), was used as the validation cohort.

### 2.2. Clinical and Pathological Characteristics

The patient's data are shown in *Supplementary Table*1, including gender, age, histological grade, tumor anatomical location, tumor type, tumor number, the presence of portal vein tumor thrombus (PVTT), cirrhosis status, tumor diameter, TNM stage, and the tumoral peritumoral expression of GRK4. The T stage was determined according to the 7th American Joint Committee on Cancer (AJCC) staging system which was used for determining the TNM stages.

### 2.3. Immunohistochemistry (IHC) Analysis

The tissue sections were dewaxed in xylene and rehydrated in gradient alcohols. The endogenous peroxidase activity was blocked with 3% H_2_O_2_ for 30 min, and the nonspecific antigen epitopes were blocked with 10% normal goat serum for 30 min. Sections were incubated with the anti-GRK4 polyclonal antibody (Sigma-Aldrich, USA, 1 : 100) overnight in a moist chamber at 4°C and then with an anti-rabbit secondary antibody (rabbit IgG H+L; Invitrogen, 1 : 5000) for 20 min. Reaction products were visualized using 3,3′-diaminobenzidine and counterstained with haematoxylin.

The tissue microarray slides were examined using a Leica CCD camera system (DM2500, Microsystems, Wetzlar, Germany). As the staining in GRK4-positive cases was distributed in most of the epithelial cells in the HCC tumoral or the paired noncancerous hepatic tissues, the immunohistochemical score was evaluated semiquantitatively by the staining intensity, which was classified as negative (score = 0), weak positive (score = 1), positive (score = 2), or strong positive (score = 3) (Supplementary Figure 1).

### 2.4. Cell Line and Lentivirus Particles

The human HCC HepG2 cell line was purchased from the Cell Bank of the Chinese Academy of Sciences (Shanghai, China). The cells were cultured in DMEM medium (Hyclone, USA) supplemented with 10% fetal bovine serum (FBS) (Gibco), 100 *μ*g/mL streptomycin, and 100 U/mL penicillin (Hyclone) at 37°C in a humidified atmosphere with 5% CO_2_. The full-length human pRK5-GRK4 plasmid was a gift from Dr. Philip B. Wedegaertner (Thomas Jefferson University, Philadelphia, PA, USA). The GRK4-overexpressing lentivirus (LV5-GRK4) and the negative control (LV5-NC) were packaged by GenePharma (Shanghai, China).

### 2.5. Western Blotting

Western blotting was performed as previously described [26]. Briefly, the cells were collected and lysed with RIPA buffer. The protein extracts were resolved on a 10% SDS-polyacrylamide denaturing gel, transferred to polyvinylidene fluoride (PVDF) (Millipore, USA) membrane, blocked, and probed with the primary antibody followed by HRP-conjugated appropriate secondary antibody. The targeted proteins were detected using an ECL reaction kit. Images were acquired and analyzed using a Bio-Rad's ChemiDoc XRS+ system (Bio-Rad Laboratories, USA).

### 2.6. MTT Assay

A MTT (3-[4,5-dimethylthiazol-2-yl]-2,5 diphenyl tetrazolium bromide, Sigma-Aldrich, USA) assay was performed as previously described [28]. Briefly, the cells were seeded in 96-well plates at a density of 4,000 cells per well. After the indicated times, 10 *μ*L of MTT (5 mg/mL, dissolved in PBS, pH 7.4) were added to each well and incubated for 4 h. The culture medium was aspirated and the plates were dried by inversion for about 15 min. The formazan crystals were then dissolved with DMSO (100 *μ*L for each well), and the absorbance was measured at 490 nm using a microplate reader (iMark, Bio-Rad Laboratories, USA).

### 2.7. Wound Healing Assay

The cells grew on 6-well plates at 90% confluence. Medium was removed and the wound was scraped off in monolayer using a 100 *μ*L pipette tip. The cells were washed with PBS for 2 times and then supplied with the fresh medium. The wells were visualized immediately (0 h) and then at 24 and 48 h after incubation at 37°C using an inverted microscope (Olympus IX81, Japan). The healing of the gaps of the monolayer was quantified by ImageJ2X software (National Institutes of Health, Bethesda, MD, USA). The migration ability of the cells was quantified by subtracting the scratch distance at 0 h from the area at 24 h and 48 h, respectively.

### 2.8. Statistical Analysis

IBM SPSS Statistics 23.0, GraphPad Prism 7.0, and EmpowerStats were used. *t*-tests or the Mann-Whitney tests (2-tailed) were used to compare continuous variables, and the *χ*^2^ test or Fisher's exact test was used to compare categorical variables. The Kaplan-Meier method and log-rank test were used to compare survival outcomes. A multivariate Cox proportional hazard regression model was used to evaluate the independent prognostic factors for OS. The *β* coefficient of the multivariate Cox regression was proportionally exchanged with a 0- to 100-point scale for the nomogram. The highest *β* coefficient was equal to 100 points. The predictive result of the nomogram was reviewed by the concordance index (C index) and calibrated with 500 bootstrap samples. Total nomogram scores of each patient were pooled, and the optimal threshold value was screened using the receiver operating the characteristic curve (ROC) by maximizing the Youden index. Sensitivity, specificity, predictive values, and likelihood ratios served to accurately determine the cut-off value of the nomogram. In all analyses, the nomogram was constructed by multivariate Cox regression with *p* < 0.1; all remaining assays were considered statistically significant at *p* < 0.05. For MTT and wound healing assays, analysis of variance (ANOVA) was used to show an overall difference between groups.

## 3. Results

### 3.1. Clinicopathological Features

Data from 448 patients who underwent hepatectomy were collected. Of these, paraffin-embedded tissues from 37 patients were inadequate for constructing the TMA, and 86 patients had received other treatment before surgery or had hepatectomy after recurrence, or survival time was less than 3 months. A total of 325 patients were included in the analysis, which contained 126 paired tumor tissues and peritumor tissues. Other 199 tumor tissues from patients that clinical information was incomplete or met other exclusion criteria were excluded from the survival analysis. They were only used for clinicopathological correlation analysis. For the validation cohort, the TMA was constructed using specimens from 93 patients, containing 87 paired paraffin-embedded HCC tissues and peritumoral tissues and six HCC tissues without paired peritumor tissues. Four patients whose OS were less than 3 months were excluded. In summary, 126 and 89 cases met the criteria for survival assays in the training cohort and validation cohort, respectively. The baseline characteristics of the patients are provided in *Supplementary Table*1. There was no significant difference in gender, tumor diameter, tumor type, grade, T stage, cirrhosis, survival status at the last follow-up, and the GRK4 expression in both tumor tissues and adjacent liver tissues between the two cohorts. The age in validation cohort patients was slightly older than that in the training cohort, while the training cohort patients showed bearing more tumors. There were more patients with portal vein tumor thrombus (PVTT) in the training cohort.

### 3.2. Immunohistochemical Findings of GRK4 in the TMA

**Figure 1:**
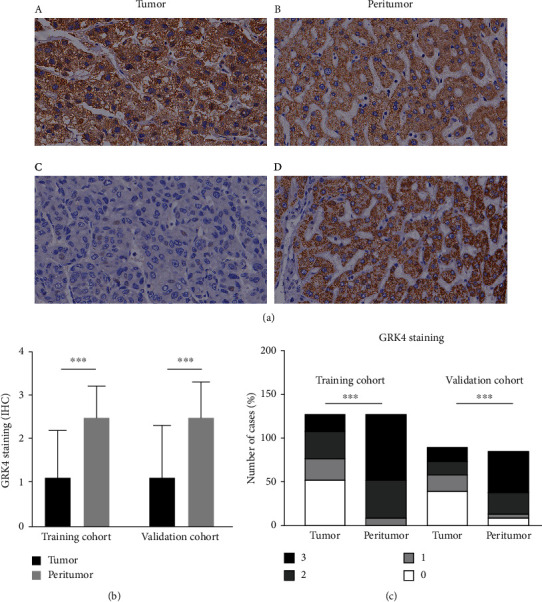
Expression of GRK4 in paired tumoral and peritumoral tissues in HCC patients. (a) Representative immunostaining of GRK4 in tumoral and peritumoral tissues of two HCC cases (×400). GRK4 is mainly expressed in the cytoplasm and membrane both in tumor and peritumor tissues. It was differentially expressed in HCC tumor tissues and rich in peritumor tissues.(A and B) GRK4 is expressed both in tumor and peritumor tissues in case 1. (C and D) GRK4 is differentially expressed in tumor and peritumor tissues in case 2. (b) Expression of GRK4 in tumor and peritumor between the training and validation cohorts. (A) Intensity of GRK4 staining was different between tumoral and peritumoral tissues. Paired sample *t*-test and independent sample *t*-test showed a statistical significance in the training cohort and validation cohort, respectively. (B) Distribution of various intensity scores of GRK4 staining in tumoral and peritumoral tissues between the cohorts. *x*^2^ test displays a significant difference between the cohorts. ^∗∗∗^*p* < 0.001.

To investigate the correlation between GRK4 expression and clinical/pathological features, we carried out IHS of the TMA. GRK4 was mainly localized cytoplasmic. The stromal cells were not immunostained (Figure 1(a)). The negative staining of GRK4 in tumoral tissues of the training and validation cohorts was 42.06% and 44.94%, respectively, while that in peritumoral tissues was 2.38% and 10.71%, respectively, which were significantly higher than those in peritumor tissues (*p* < 0.001, *Supplementary Table*1). The average GRK4 intensity scores in tumor and peritumor in the training cohort were 1.1 and 2.5, respectively (*p* < 0.001), and those in the validation cohort were 1.1 and 2.3 (*p* < 0.001), respectively (Figure 1(b)). More than 90% of patients whose peritumor tissues displayed positive expression of GRK4.

### 3.3. Correlations between GRK4 Staining and Clinicopathological Features

As shown in *Supplementary Table*2, there was no significant correlation between the expression of GRK4 in or around the tumors and the pathological features in the training or validation cohorts. Neither tumoral nor peritumoral GRK4 expression differed significantly between patients with high and low histopathological grade (*p* = 0.36), PVTT (*p* = 0.13), cirrhosis (*p* = 0.87), number of tumors (*p* = 0.95), or T stage (*p* = 0.372). As expected, most of the surviving patients were not those who lacked GRK4 expression (*p* < 0.05) by the last follow-up in both cohorts. Interestingly, half of the patients with high GRK4 expression in the peritumor tissue displayed nonstaining in tumor tissues in the training cohort (*p* = 0.05). However, a different outcome was observed after information of the additional 199 cases without the paired peritumor tissues was included. Therefore, the data were reclassified into two clusters on the basis of low (score 0-1) and high (score 2-3) tumoral GRK4 expression. Meanwhile, the other data, such as T stage, were reassayed and merged. The results showed that the expression of GRK4 in tumors was correlated with histological grade (*p* < 0.001), T stage (*p* < 0.001), N stage (*p* < 0.05), and the TNM stage (*p* < 0.001) (Table 1).

### 3.4. Prognosis and Independent Prognostic Factors

The median follow-up time was 35 months (range of 10–52 months) in the training cohort and 79 months (range of 11–80 months) in the validation cohort. In the training cohort, the 1-, 3-, and 5-year survival rates were 89%, 42%, and 16%, respectively, and the corresponding rates for the validation cohort were 76%, 42%, and 38%. The median survival time was 32 months in both cohorts (*p* = 0.21) (Supplementary Figure 2).

All parameters displayed in the *Supplementary Table*1 were analyzed by the univariate Cox regression. The results are reported in Table 2. T stage, expression intensity of intratumoral GRK4, tumor type, tumor diameter, number of tumors, and presence of PVTT were associated with OS (Supplementary Figure 3). Interestingly, intratumoral GRK4 overexpression was identified as a protective factor. The peritumoral GRK4 was generally independent of OS, although it was highly expressed in both cohorts.

### 3.5. Construction and Validation of a GRK4-Based Predictive Nomogram

**Figure 2:**
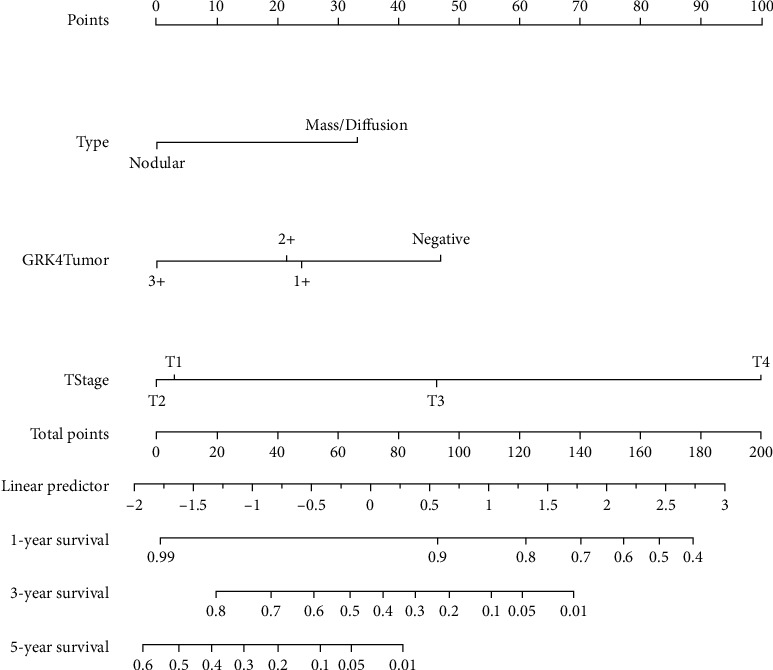
Nomogram to estimate the risk for 1-, 3-, and 5-year OS, combining GRK4 presence in tumor tissue, tumor type, and T stage to form the nomogram. To use the nomogram, find the position of each variable on the corresponding axis, draw a line to the point value axis to determine the number of points for each variable, add the points from all of the variables, and draw a line from the total point axis to determine the probabilities at the lower line of the nomogram.

The results of the multivariate Cox regression analysis are shown in Table 3. T stage (hazard ratio (HR) 2.135, 95% confidence interval (95% CI) 1.520–3.000, *p* < 0.01), tumor type (HR 2.10, 95% CI 0.88–5, *p* < 0.1), and intratumoral GRK4 (HR 0.53, 95% CI 0.26–1.11, *p* < 0.05) were determined to be independent risk factors, and they were incorporated into a nomogram to estimate the risk for OS (Figure 2). The nomogram performed well in the exact evaluation of the risk of 5-year OS with an AUC of 0.82, which was further assessed by the index of concordance (C-index) and displayed ideal consistency for OS risk assessed with a C-index 0.82 (95% CI 0.74–0.90) by internal validation using bootstrap sampling (Supplementary Figure 4a). For the external validation, the nomogram determined a C-index of 0.77 (95% CI 0.68–0.87) for appraising the risk of OS, and the calibration plot presents good concordance (Supplementary Figure 4b).

### 3.6. Risk for OS Derived from Nomogram Scores

**Figure 3:**
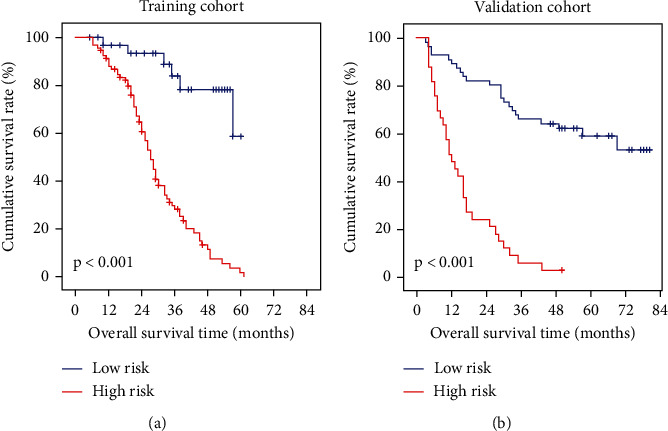
Comparison of cumulative OS curves between of training cohort and validation cohort after risk stratification. (a) OS curves of subgroups with risk stratified by dividing HCC patients into low-risk and high-risk groups based on the nomogram score cut-off value of 32 in the training cohort, which was statistically significant (*p* < 0.001). (b) OS plots of the subgroups with risk stratified by cut-off value of 78 in the validation cohort show significant differences (*p* < 0.001).

The best threshold value of the overall nomogram scores was found to be 32 and 78 in the training and validation cohorts, respectively. The sensitivity, specificity, positive predictive value, and negative predictive value in the training cohort were 86%, 70%, 83%, and 75%, respectively, and the presence was 44%, 100%, 100%, and 52% in the validation cohort, respectively (Table 4). Risk stratification was carried out using the cut-off value and cases were reclassified low risk (≤cut-off value) and high risk (>cut-off value). Risk stratification exhibited excellent discrimination compared with any of the independent prognostic factors (Figure 3).

### 3.7. Overexpression of GRK4 Inhibits HCC Cell Proliferation and Migration

**Figure 4:**
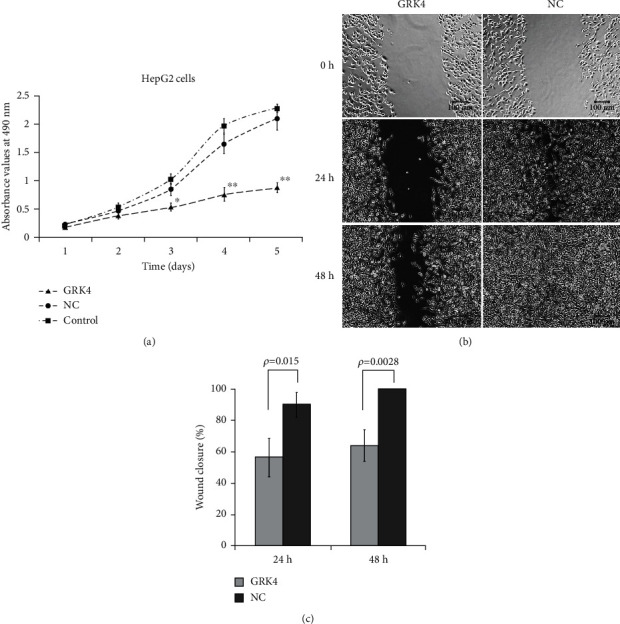
GRK4 overexpression inhibits proliferation and migration of HepG2 cells. The cells were infected with lentivirus-GRK4 vector (LV5-GRK4) or control vector (LV5-NC) for 48 h and then seeded at 1 × 10^5^ cell per well onto 96-well plate. (a) The effect of GRK4 on cell proliferation was analyzed by MTT assay after 24, 48, 72, 96, and 120 h. The results are representative of mean ± SD of three independent experiments (^∗^*p* < 0.05 and ^∗∗^*p* < 0.01 vs. uninfected control). (b) The migration ability of cells was evaluated by wound healing assay. Images were taken immediately and also 24 and 48 h after creating the scratch using an inverted microscope. (c) Quantitative data of the healing of the gaps of the monolayer were shown. Each bar represents the mean ± SD of three independent experiments.

To examine the biological functions of GRK4 in HCC, we, respectively, transfected the human HCC HepG2 cells with GRK4-GFP and negative control (NC)-GFP lentiviruses. The lentiviral infection efficiency was >90% at 48 h after infection and the protein levels were determined by western blotting (Supplementary Figure 5). The cell growth curve and migration ability were, respectively, measured by MTT assay and the wound healing assay. Both the proliferation and motility capabilities were significantly decreased in the cells infected with the lentivirus overexpressing GRK4, compared to cells infected with the NC lentivirus (Figure 4). These results indicate that overexpression of GRK4 was capable of restraining the proliferation and migration abilities of the hepatocellular carcinoma cells.

## 4. Discussion

GRKs participate in a wide range of cellular physiological and pathological activities. There are increasing evidences indicated that some of which are closely related to the occurrence and progression of tumors [14, 15]. In the present study, we reported that absent expression of GRK4 in tumors was significantly associated with poor outcomes in HCC patients (*p* < 0.05). More than 30% of patients lacked GRK4 expression in tumors. Peritumoral GRK4 expression was not associated with OS. This study also indicates that a combination of tumor type (mass or diffusion) and T stage provides more power to predict patient outcomes. To our knowledge, this is the first study that unveils the GRK4 distribution in HCC tumors as well as its prognostic significance.

The intensity scores of GRK4 staining in the cytoplasm were lower in HCC tissue than that in peritumor tissue. The differential expression in peritumor and tumor tissues might suggest GRK4 a critical factor for OS in HCC patients. There are increasing evidences supporting that activation of the GPCR signaling results from autocrine and paracrine signals as well as aberrant GPCR overexpression in tumor cells, facilitating angiogenesis and metastasis [29, 30]. GRKs negatively regulate GPCR signalings and are involved in neoplasia development. For example, downregulation of GRK2, GRK3, GRK5, and GRK6 promoted tumorigenesis and metastasis in Kaposi sarcoma, basal-like breast cancer, colon cancer, and medulloblastoma [17, 31–34]. In granulosa cell tumors (GCT), lower expression of GRK4*α*/*β* was observed in malignant tumors than in nonmalignant tumors, whereas GRK4*γ*/*δ* expression was observed in all tumor samples, and GRK4 isoforms may weaken follicle-stimulating hormone receptor uncoupling and desensitization in the pathogenesis of GCT [24]. GRK4 was overexpressed in hyperfunctioning thyroid nodules (HTNs) compared with their adjacent tissues but failed to induce thyroid-stimulating hormone receptor (TSHR) desensitization, which may cause HTN [35]. GRK4 was also found overexpressed in invasive breast cancer and frequently mutated in high microsatellite instability (MSI-H) colorectal tumors [25, 36]. These observations suggest a link of GRK4 to tumor biology.

The expression pattern and function of the GRK family members have been reported differed among various tumors [14, 15, 33, 34]. In HCC, GRK3 was expressed in larger tumors and early stage diseases, whereas GRK6 was prone to expression in smaller, moderate histological grade and metastatic tumors [17, 18]. In this study, we found that tumoral GRK4 was connected to histological grade, T stage, N stage, and total stage; GRK4 tended to be expressed in patients who had early stage HCC with no lymph node involvement (*Supplementary Table*1).

We identified that a lack of GRK4 expression was an independent risk factor for HCC patients. In HCC, more than five staging systems are applied in practice, and each system has a particularly useful window [37]. Interestingly, regardless of which staging system was applied, almost every system was linked to the prognosis in a different study [37, 38]. We showed that an improving nomogram of tumoral GRK4 expression plus tumor type and T stage performed well with the AUCs of 0.82 in predicting the prognosis of HCC patients. In this nomogram model, the performance of T stage (T1 or T2) or tumoral GRK4 intensity (score 1 or score 2) alone did not perform well. However, the analysis combined with the three factors provides unexpected benefits (Figure 3). For the clinical practice of the model, cut-off values of 32 and 78 were used in the training and validation cohorts, respectively. These values were convenient and precise since they were calculated by sensitivity, specificity, positive predictive value, and negative predictive value. According to the predictive results, the nomogram might serve as a useful tool to determine whether patients will benefit from hepatectomy or clinical trials.

We previously reported that GRK4 is capable of inducing cellular senescence in HEK293 cells [26], and overexpression of GRK4 affects pathways of cell development, proliferation, and cell death [27]. However, the specific effects of GRK4 on cancer cells are unclear. Here, we showed that overexpression of GRK4 suppressed the proliferation and migration abilities of human HCC HepG2 cells, which would partially explain the above observation that HCC patients with high expression of GRK4 in tumor tissues have a better prognosis. The molecular mechanism underlying is worth further study.

## 5. Conclusions

The GRK4 is differentially expressed in HCC tumor tissues. The low expression of GRK4 in tumor is associated with poor OS in HCC patients. A novel nomogram model with tumoral GRK4 expression provides a better practical tool in assessment of OS risk. GRK4 overexpression inhibits proliferation and migration of HCC cells.

## Figures and Tables

**Table 1:** Correlation between GRK4 expression and clinicopathological features in HCC 325 patients.

Features	Case no. (*n*)	GRK4 expression		*χ* ^2^	*p* value
		High (*n*, %)	Low (*n*, %)		
Sex				0.44	0.559
Female	60	21 (16.7)	39 (19.6)		
Male	265	105 (83.3)	160 (80.4)		
Age (years)				2.03	0.172
<50	158	55(43.7)	103(51.8)		
≥50	167	71 (56.3)	96 (48.2)		
Histological grade				81.357	<0.001
I	60	54 (42.9)	6 (3.0)		
II-IV	265	72 (57.1)	193 (97)		
T stage				21.436	<0.001
T1-T2	225	106 (84.1)	119 (59.8)		
T3-T4	100	20 (15.9)	80 (40.2)		
N stage				4.061	0.044
N0	305	123 (97.6)	182 (91.5)		
N1	20	3 (2.4)	17 (8.5)		
M stage				1.689	0.194
M0	313	124 (98.4)	189 (95)		
M1	12	2 (1.6)	10 (5)		
TNM stage				18.964	<0.001
I-II	208	99 (78.6)	109 (54.8)		
III-IV	117	27 (21.4)	90 (45.2)		

**Table 2:** Univariate Cox regression analysis of factors associated with survival.

Variable	HR (95% CI)	*p* value
Age	0.99 (0.97, 1.01) 0.2403	0.24
Cirrhosis, no vs. yes	0.94 (0.67, 1.31) 0.7015	0.70
Tumor diameter	1.06 (1.03, 1.10)	0.00
Venous invasion, no vs. yes	2.39 (1.63, 3.49)	0.00
Tumor type, nodular vs. diffuse	2.97 (2.06, 4.27)	0.00
Number of tumors, solitary vs. multiple	2.39 (1.60, 3.58)	0.00
Anatomic location, left vs. right^∗^	0.69 (0.49, 0.99) 0.0444	0.04
T stage		
2 vs. 1	1.19 (0.68, 2.08) 0.5447	0.54
3 vs. 1	4.40 (2.85, 6.81)	0.00
4 vs. 1	10.87 (5.13, 23.03)	0.00
Tumor GRK4		
1+ vs. negative	0.68 (0.43, 1.05) 0.0837	0.08
2+ vs. negative	0.60 (0.37, 0.98) 0.0413	0.04
3+ vs. negative	0.28 (0.15, 0.52)	0.00
Peritumor GRK4		
1+ vs. negative	1.74 (0.56, 5.40) 0.3397	0.34
2+ vs. negative	1.42 (0.60, 3.34) 0.4268	0.43
3+ vs. negative	1.70 (0.74, 3.91) 0.2125	0.21
Grade		
2 vs. 1	1.10 (0.65, 1.87) 0.7218	0.72
3 vs. 1	1.40 (0.71, 2.75) 0.3293	0.33

**Table 3:** Multivariate Cox regression analysis of factors associated with survival.

Variable	Multivariate	
	HR (95% CI)	*p* value
Tumor diameter	1.04 (0.96, 1.13)	0.31
Venous invasion, no vs. yes	1.07 (0.52, 2.20)	0.85
Tumor type, diffuse vs. nodular	2.10 (0.88, 5.00)	0.09
Number of tumors, solitary vs. multiple	0.89 (0.41, 1.94)	0.77
Anatomic location, left vs. right^∗^	0.94 (0.57, 1.56)	0.82
T stage		
1 vs. 3	0.77 (0.32, 1.85)	0.04
2 vs. 3	2.81 (1.02, 7.72)	0.01
4 vs. 3	10.75 (2.57, 44.97)	0.02
Tumor GRK4		
1+ vs. negative	0.50 (0.26, 0.97)	0.04
2+ vs. negative	0.53 (0.26, 1.11)	0.09
3+ vs. negative	0.28 (0.12, 0.64)	0.00

**Table 4:** Accuracy of the prediction score of the nomogram for estimating the risk of OS.

Variable	Training cohort (95% CI)			Validation cohort (95% CI)		
AUC	0.82	0.74	0.90	0.77	0.68	0.87
Cutoff score	32			78		
Sensitivity (%)	86	72	88	44	32	71
Specificity (%)	70	47	72	100	32	100
Positive predictive value (%)	83	75	83	100	69	100
Negative predictive value (%)	75	61	86	52	52	70
Positive likelihood ratio	2.89	2.12	3.26	1	1.36	2.34
Negative likelihood ratio	0.2	0.23	0.43	inf	0.56	0.46

## Data Availability

The data that support the findings of this study are available from the corresponding author (XJ), upon reasonable request.

## Abbreviations

GPCR: G protein-coupled receptor
GRK: G protein-coupled receptor kinase
HCC: Hepatocellular carcinoma
OS: Overall survival
C-index: Concordance index
RFA: Radiofrequency ablation
TACE: Transcatheter arterial chemoembolization
TMA: Tissue microarray
PVTT: Portal vein tumor thrombus
IHC: Immunohistochemistry
ROC: Receiver operating characteristic curve
AUC: Area under the ROC curve.

## Additional Points

*Study Highlights.* What is known. (i) Hepatocellular carcinoma (HCC) is the third leading cause of cancer-related mortality worldwide. (ii) Prediction of the prognosis for postoperative HCC patients remains a challenge. (iii) G protein-coupled receptor kinase (GRK) 4 inhibits cell proliferation and potentially suppresses cancer progression. What is new here. (i) GRK4 is differentially expressed in HCC tumor tissues. (ii) Low expression of GRK4 is associated with the poor prognosis. (iii) A nomogram including tumoral GRK4 expression improves the predictive accuracy of overall survival in HCC patients. (iv) A novel inhibitory effect of GRK4 on HCC cell proliferation and motility.

## Supplementary Materials

Supplementary Materials Supplementary Table 1: baseline characteristics in both cohorts. Supplementary Table 2: correlation of tumor GRK4 and clinicopathological characteristics. Supplementary Table 3: correlation of peritumor GRK4 and clinicopathological characteristics in both cohorts. Supplementary Figure 1: representative results of GRK4 immunostaining in HCC tumoral and peritumoral tissues (×400). (a and e) Negative GRK4 staining in tumor cells or few positive cells in peitumoral tissue (score: 0). (b and f) Weak cytoplasmic/membranous staining in <50% of cells (score: 1). (c and g) Weak cytoplasmic/membranous staining in >50% of cells or cells with moderate immunoreactivity (score: 2). (d and h) Strong cytoplasmic/membranous immunoreactivity in cells (score: 3). Supplementary Figure 2: comparison of cumulative OS curves between the training cohort and validation cohort. The log-rank test of the OS curves of patients with HCC in the training set and validation set showed that the OS was not significantly different between the two cohorts. Supplementary Figure 3: univariate Cox regression analysis of six factors associated with survival in the training and validation cohorts. Univariate analysis showed that tumor T stage, intratumoral GRK4, tumor type, tumor diameter, number of tumors, and presence of PVTT were associated with OS in both cohorts. Left, training cohort; right, validation cohort. Supplementary Figure 4: the calibration plot of nomogram. (a) The calibration plot shows the prediction performance of the proposed nomogram in the training cohort (*n* = 126). (b) Validity of the predictive performance of the nomogram in estimating the risk of OS in the validation cohort (*n* = 89). The curve closer to the 45° line (blue line) indicates the better performance for both (a) and (b). Supplementary Figure 5: lentiviral-mediated GRK4 overexpression in HepG2 cells. HepG2 cells were infected with lentivirus-GRK4 vector (LV5-GRK4) or control vector (LV5-NC) for 48 h. (A) The transfection efficiency of the lentiviruses was detected under a fluorescence microscope and the percentage of GFP-positive cells was more than 90%. (B) Total proteins were extracted and western blot analysis was performed to determine the expression of GRK4 in cells. Actin was shown as a loading control. (C) Quantitative data of the protein levels were shown. Each bar represents the mean ± SD of three independent experiments (^∗∗^*p* < 0.01 vs. LV5-NC).
