# Supplementary material for: G Protein-Coupled Receptor Kinase 4 Is a Novel Prognostic Factor in Hepatocellular Carcinoma
Source: Dis Markers. 2022 Jun 20;2022:2628879. doi: 10.1155/2022/2628879 (PMC9236775; doi:10.1155/2022/2628879)
Supplement: Supplementary Materials — Supplementary Table 1: baseline characteristics in both cohorts. Supplementary Table 2: correlation of tumor GRK4 and clinicopathological characteristics. Supplementary Table 3: correlation of peritumor GRK4 and clinicopathological characteristics in both cohorts. Supplementary Figure 1: representative results of GRK4 immunostaining in HCC tumoral and peritumoral tissues (×400). (a and e) Negative GRK4 staining in tumor cells or few positive cells in peitumoral tissue (score: 0). (b and f) Weak cytoplasmic/membranous staining in <50% of cells (score: 1). (c and g) Weak cytoplasmic/membranous staining in >50% of cells or cells with moderate immunoreactivity (score: 2). (d and h) Strong cytoplasmic/membranous immunoreactivity in cells (score: 3). Supplementary Figure 2: comparison of cumulative OS curves between the training cohort and validation cohort. The log-rank test of the OS curves of patients with HCC in the training set and validation set showed that the OS was not significantly different between the two cohorts. Supplementary Figure 3: univariate Cox regression analysis of six factors associated with survival in the training and validation cohorts. Univariate analysis showed that tumor T stage, intratumoral GRK4, tumor type, tumor diameter, number of tumors, and presence of PVTT were associated with OS in both cohorts. Left, training cohort; right, validation cohort. Supplementary Figure 4: the calibration plot of nomogram. (a) The calibration plot shows the prediction performance of the proposed nomogram in the training cohort (n = 126). (b) Validity of the predictive performance of the nomogram in estimating the risk of OS in the validation cohort (n = 89). The curve closer to the 45° line (blue line) indicates the better performance for both (a) and (b). Supplementary Figure 5: lentiviral-mediated GRK4 overexpression in HepG2 cells. HepG2 cells were infected with lentivirus-GRK4 vector (LV5-GRK4) or control vector (LV5-NC) for 48 h. (A) The transfection e [file 2628879.f1.zip › 2628879.f1/Supplementary Table 2.pdf]

Supplementary Table 2. Correlation of tumor GRK4 and clinicopathological characeristics

	Training Cohort					Validation Cohort				
	negative	1+	2+	3+	*P*-value	negative	1+	2+	3+	*P*-value
N	53	25	30	18		40	20	13	16	
Age	49.36 ± 11.26	48.40 ± 8.97	53.90 ± 10.26	50.39 ± 7.72	0.17	52.02 ± 9.58	55.30 ± 9.02	55.85 ± 13.23	54.62 ± 9.66	0.51
Tumor diameter	8.62 ± 3.88	9.24 ± 3.87	8.00 ± 3.60	9.89 ± 4.44	0.38	7.25 ± 4.54	7.35 ± 4.55	7.58 ± 6.09	9.06 ± 7.94	0.72
Status					<0.01					0.04
Alive	8 (15.09%)	10 (40.00%)	18 (60.00%)	11 (61.11%)		11 (27.50%)	8 (40.00%)	4 (30.77%)	11 (68.75%)	
Dead	45 (84.91%)	15 (60.00%)	12 (40.00%)	7 (38.89%)		29 (72.50%)	12 (60.00%)	9 (69.23%)	5 (31.25%)	
Gender					0.40					0.58
Male	43 (81.13%)	23 (92.00%)	26 (86.67%)	17 (94.44%)		34 (85.00%)	19 (95.00%)	12 (92.31%)	15 (93.75%)	
Female	10 (18.87%)	2 (8.00%)	4 (13.33%)	1 (5.56%)		6 (15.00%)	1 (5.00%)	1 (7.69%)	1 (6.25%)	
Anatomic location					0.98					0.20
Left or other	24 (45.28%)	11 (44.00%)	14 (46.67%)	9 (50.00%)		3 (7.50%)	4 (20.00%)	4 (30.77%)	3 (18.75%)	
Right	29 (54.72%)	14 (56.00%)	16 (53.33%)	9 (50.00%)		37 (92.50%)	16 (80.00%)	9 (69.23%)	13 (81.25%)	
Number of tumors					0.95					0.98
Solitary	38 (71.70%)	18 (72.00%)	21 (70.00%)	14 (77.78%)		36 (90.00%)	18 (90.00%)	12 (92.31%)	14 (87.50%)	
Multiple	15 (28.30%)	7 (28.00%)	9 (30.00%)	4 (22.22%)		4 (10.00%)	2 (10.00%)	1 (7.69%)	2 (12.50%)	
Tumor type					0.43					0.97
Nodular	24 (45.28%)	9 (36.00%)	17 (56.67%)	7 (38.89%)		23 (57.50%)	12 (60.00%)	7 (53.85%)	10 (62.50%)	
Mass or diffuse	29 (54.72%)	16 (64.00%)	13 (43.33%)	11 (61.11%)		17 (42.50%)	8 (40.00%)	6 (46.15%)	6 (37.50%)	
Cirrhosis					0.87					0.37
No	23 (43.40%)	12 (48.00%)	15 (50.00%)	7 (38.89%)		23 (57.50%)	10 (50.00%)	6 (46.15%)	12 (75.00%)	
Yes	30 (56.60%)	13 (52.00%)	15 (50.00%)	11 (61.11%)		17 (42.50%)	10 (50.00%)	7 (53.85%)	4 (25.00%)	
Venous invasion					0.13					0.50
No	39 (73.58%)	13 (52.00%)	24 (80.00%)	13 (72.22%)		35 (87.50%)	19 (95.00%)	10 (76.92%)	14 (87.50%)	
Yes	14 (26.42%)	12 (48.00%)	6 (20.00%)	5 (27.78%)		5 (12.50%)	1 (5.00%)	3 (23.08%)	2 (12.50%)	
Grade					0.36					0.16

1	5 (9.43%)	7 (28.00%)	5 (16.67%)	2 (11.11%)		1 (2.50%)	2 (10.00%)	1 (7.69%)	3 (18.75%)	
2	42 (79.25%)	15 (60.00%)	20 (66.67%)	15 (83.33%)		31 (77.50%)	17 (85.00%)	12 (92.31%)	10 (62.50%)	
3	6 (11.32%)	3 (12.00%)	5 (16.67%)	1 (5.56%)		8 (20.00%)	1 (5.00%)	0 (0.00%)	3 (18.75%)	
T Stage					0.37					0.45
1	19 (35.85%)	5 (20.00%)	13 (43.33%)	5 (27.78%)		13 (32.50%)	7 (35.00%)	8 (61.54%)	7 (43.75%)	
2	10 (18.87%)	6 (24.00%)	7 (23.33%)	7 (38.89%)		7 (17.50%)	4 (20.00%)	1 (7.69%)	3 (18.75%)	
3	23 (43.40%)	12 (48.00%)	8 (26.67%)	6 (33.33%)		19 (47.50%)	8 (40.00%)	2 (15.38%)	5 (31.25%)	
4	1 (1.89%)	2 (8.00%)	2 (6.67%)	0 (0.00%)		1 (2.50%)	1 (5.00%)	2 (15.38%)	1 (6.25%)	
GRK4 peritumor					**0.05**					0.26
negative	0 (0.00%)	1 (4.00%)	1 (3.33%)	1 (5.56%)		5 (13.16%)	2 (10.53%)	0 (0.00%)	2 (13.33%)	
1+	3 (5.66%)	1 (4.00%)	0 (0.00%)	2 (11.11%)		1 (2.63%)	2 (10.53%)	0 (0.00%)	1 (6.67%)	
2+	19 (35.85%)	5 (20.00%)	8 (26.67%)	11 (61.11%)		9 (23.68%)	9 (47.37%)	2 (16.67%)	5 (33.33%)	
3+	31 (58.49%)	18 (72.00%)	21 (70.00%)	4 (22.22%)		23 (60.53%)	6 (31.58%)	10 (83.33%)	7 (46.67%)	
